# Mental illness and health literacy in people with cancer: the Australian National Health Survey analysis

**DOI:** 10.1007/s00520-026-10962-x

**Published:** 2026-07-02

**Authors:** Huah Shin Ng, Cartier Dods, Jason Dimian, Richard Woodman, Lisa Beatty

**Affiliations:** 1 Flinders Health and Medical Research Institute, College of Medicine and Public Health, Flinders University, Sturt Road, Bedford Park, SA 5042 Australia; 2 SA Pharmacy, Northern Adelaide Local Health Network, SA Health, Adelaide, SA Australia; 3 Flinders University Institute for Mental Health and Wellbeing, Psychology and Social Work, College of Education, Flinders University, Adelaide, SA Australia

**Keywords:** Cancer, Mental illness, Health literacy, Cross-sectional, Australia

## Abstract

**Purpose:**

To examine factors associated with mental illness (MI) in people with and without cancer, and the associations between cancer, MI and health literacy (HL).

**Methods:**

This cross-sectional study was conducted using data from the Australian National Health Survey 2017–2018 and HL Survey. All participants aged ≥ 25 years were categorised into four groups based on self-reported cancer and MI. Multivariable logistic regressions were performed to examine characteristics associated with MI, with stratification by cancer status. General linear models were used to assess the association between nine individual HL domain scores and cancer and MI.

**Results:**

About 30% of people with cancer (*n* = 690/2276) and 27% of those without cancer (*n* = 3483/12920) reported having a MI. Several characteristics were associated with higher odds of MI in both cancer and non-cancer groups: female gender, younger age, obesity, currently smoking, residing in a major city, lower socioeconomic status, having more comorbidities and being physically less active. For most of these characteristics, the associated risk was higher in people with cancer. MI was negatively associated with seven individual HL domain scores, whilst having cancer was negatively associated with only one domain.

**Conclusion:**

Risk factors for MI appeared to be similar in persons with and without cancer, but the associated relative risks are higher in those with cancer. HL is negatively associated with both MI and cancer, with stronger associations for MI. Improving HL in those with MI and cancer has the potential to improve modifiable risk factors and outcomes of this population.

**Supplementary Information:**

The online version contains supplementary material available at 10.1007/s00520-026-10962-x.

## Introduction

Cancer survival rates have been improving owing to advancements in cancer treatments and timely diagnosis, with current Australian 5-year survival rates sitting at 90% across some cancer types [1]. However, these benefits do not apply to all universally, with certain subgroups experiencing disproportionately inferior outcomes, such as higher mortality rates and poorer health status [2, 3]. One such group is those with comorbid mental illness (MI) [4, 5]. People living with MI is now a recognised priority population in the Australian Cancer Plan (a ten-year plan designed to improve cancer outcomes in Australia especially in those with poorer health outcomes) [6].

MI is highly prevalent in people with cancer [7], with a documented higher prevalence rate than the general population [8]. For example, depression–one of the most common mental disorders–has a prevalence of about 24% among cancer survivors [7], compared to 4% in the general population [9]. A systematic review showed that several characteristics were associated with increased risk of depression in people with cancer, including sociodemographic (e.g., lower education and lower income), lifestyle (e.g., smoking) and medical factors (e.g., comorbidities) [10]. However, results varied across included studies, and the generalisability of the findings remains to be explored. There is limited population-level research in the Australian setting that have examined the differences in risk factors of MI amongst the cancer and non-cancer populations to inform the development of targeted intervention strategies.

One potential mechanism driving the differences in health outcomes between those with versus those without MI and cancer could be health literacy (HL). Defined as the cognitive and social skills which determine an individual’s ability to understand, access and use information regarding good health practices to make informed decisions [11], low HL in general has been linked to higher risks of hospitalisation rates, mortality and higher prevalence of modifiable risk factors (e.g., smoking, overweight, insufficient physical activity) in the general population [12, 13]. HL is shaped by the interaction between an individual’s skills and the demands and complexity of the healthcare system and organisations in providing accessible and understandable information and services tailored to population needs [14]. Among people with cancer, previous research suggest that low HL is associated with poorer quality of life, inferior experience of care and greater difficulties in understanding and processing cancer-related information [15], while among those with MI, low HL results in greater difficulties in engaging with health professionals and a longer hospitalisation [16]. However, a clear limitation of the research on HL to date is that it has largely focused on cancer and MI independently [15, 17, 18], with limited data on the association between the coexistence of both conditions and HL.

Therefore, the aims of the study were to: (i) determine risk factors associated with MI in people with and without cancer, (ii) examine whether the strength of the associations between risk factors and MI differs in those with and without cancer, and (iii) assess the relationship between MI status and HL in those with and without cancer.

## Methods

### Data source

Data from the Australian National Health Survey (NHS) 2017–2018, conducted by the Australian Bureau of Statistics (ABS), that collected a range of health-related information including health behaviours, risk factors and disease prevalence in Australia [19] was used in this study. The standardised survey was administered by trained ABS interviewers through fully structured face-to-face interviews in a representative sample of 16,376 private dwellings (e.g., houses, home units, flats, caravans and other structures being used as a place of residence) from across Australian states and territories (response rate: 76%) [19]. Data was recorded electronically at the point of interview by the interviewers. De-identified basic unit records (microdata) was obtained from the ABS to perform the cross-sectional analysis.

### Cancer status

All participants of the NHS 2017–2018 aged ≥ 25 years were identified and categorised into two groups based on self-reported history of cancer (cancer versus non-cancer group). The cancer group included any reported cancer including malignant neoplasms of other sites or site unknown and/or skin malignant neoplasms that are current or in remission. All other participants without a history of cancer were included in the non-cancer group.

### Mental illness

Participants were asked if they have any long-term health conditions including mental and behavioural problems that have lasted or expected to last for 6 months or longer. Mental and behavioural problems that were listed in the survey were each classified based on International Classification of Diseases-10 codes and included organic mental problems (e.g., dementia including Alzheimer’s disease), alcohol and drug problems (e.g., harmful use or dependence on alcohol, medicinal/prescription drugs or drugs), mood [affective] disorders (e.g., feeling depressed, depression, manic episode, bipolar affective disorder), anxiety-related problems (e.g., anxiety disorder including generalised anxiety disorders, panic disorder, obsessive compulsive disorder, post-traumatic stress disorder), problems of psychological development (e.g., autism spectrum disorders such as Rett’s syndrome and Asperger syndrome), behavioural, cognitive and emotional problems with usual onset in childhood/adolescence (e.g., attention deficit hyperactivity disorder, conduct disorders children/adolescence), other mental and behavioural problems (e.g., schizophrenia related conditions, conduct disorders), and other symptoms, signs involving cognition perceptions, emotional state and behaviours [19]. Participants who self-reported having any mental and behavioural problems as a ‘current’ condition were identified and grouped in the ‘MI’ group. The remaining participants who reported either a prior history of MI (‘not current’), or no history MI condition made up the ‘no MI’ group.

### Sociodemographic, lifestyle and comorbidity variables

Sociodemographic data extracted included: sex, age, country of birth, geographical location, highest education attainment, and socioeconomic status measured by index of relative socioeconomic disadvantage according to geographic areas [19]. Lifestyle risk factors extracted included: smoker status, level of all physical activity undertaken at workplace and exercise combined in the last week (very low, low, moderate or high level, no exercise, and not stated), alcohol consumption (lifetime alcohol risk level based on the 7-day average consumption using 2009 National Health and Medical Research Council guidelines), dietary intake (whether respondents were meeting fruit and vegetable consumption recommendations according to 2013 National Health and Medical Research Council Australian dietary guidelines), and body mass index [19].

The number of other current long-term diseases (‘comorbidity’) was calculated according to 11 broad disease groupings including diseases of circulatory, digestive, endocrine, genitourinary, musculoskeletal, nervous, and respiratory systems, diseases of blood, skin and eye, and certain infections [2]. Both cancer and MI were excluded in the count of the number of diseases.

### Health literacy

The HL Survey was conducted in the sub-group of NHS participants aged ≥ 18 years, with 5790 fully responding adults [20]. Of these, 5450 aged ≥ 25 years were included in our analysis examining the effects of cancer and MI status on HL. We excluded adolescent and young adults (described as 15–24 years in Australia) [21] as their healthcare needs may differ from those in adults. Briefly, the HL Survey used the HL Questionnaire [11] that comprises 44 questions (items) covering nine independent domains of HL: (1) feeling understood and supported by healthcare providers; (2) having sufficient information to manage my health; (3) actively managing my health; (4) social support for health; (5) appraisal of health information; (6) ability to actively engage with healthcare providers; (7) navigating the healthcare system; (8) ability to find good health information; and (9) understand health information well enough to know what to do. Data was collected for each item using either four-point responses for HL statements (‘strongly agree’, ‘agree’, ‘disagree’ or ‘strongly disagree’ for domains 1 to 5), or five-response options for the perceived problems on the characteristics of HL (‘always easy’, ‘usually easy’, ‘sometimes difficult’, ‘usually difficult’ or ‘cannot do or always difficult’ for domains 6 to 9) [11, 20]. Nine separate scores were generated (one for each domain) [22]. The mean item score for each domain was obtained by dividing the sum of item scores with the number of items (ranging from 4 to 6) in that individual domain. A higher score indicated a higher HL.

### Statistical analysis

Descriptive statistics were used to summarise the characteristics of the study population by cancer and MI status. Separate multivariable logistic regression analyses were performed to examine which sociodemographic (sex, age, country of birth, geographical location, education level, socioeconomic status), lifestyle (smoker status, physical activity, alcohol consumption, dietary intake, body mass index) and comorbidity characteristics were associated with MI (yes versus no) in cancer and non-cancer groups. For the cancer group, cancer disease status (current cancer or non-current cancer) was included as an additional variable in the multivariable logistic regression model. The results were reported as adjusted odds ratio (aOR) with 95% confidence interval.

General linear models were conducted to assess the associations between cancer and MI and each HL domain. Models were adjusted for the same sociodemographic, lifestyle factors and comorbidities listed as potential predictors in the logistic regression analysis as described above. Separate general linear models were conducted for the cancer group to examine the associations between cancer disease status (current versus non-current cancer) and MI and each HL domain.

### Ethical consideration

We obtained ethics approval from the Flinders University Human Ethics Low Risk Panel (#9083). The study was conducted in accordance with the ethical standards of the 1964 Declaration of Helsinki and its later amendments.

## Results

### Cohort characteristics

The study included a total of 15,196 people aged ≥ 25 years (*n* = 2276, 15% with cancer; *n* = 12,920, 85% without cancer). There was a significant difference (*p* = 0.0009) in the MI disease prevalence between the two groups; nearly one-third of people with cancer (*n* = 690; 30%) vs. over one-quarter of those without cancer (*n* = 3483; 27%) reported having a current MI (Table 1). A significantly higher proportion of people with cancer were older (57% vs. 22% aged ≥ 65 years), born in Australia (79% vs. 67%), resided outside of major cities (44% vs. 38%), were an ex-/current smoker (56% vs. 49%) and overweight/obese (75% vs. 69%), had no non-school qualification (42% vs. 34%), no physical activity (23% vs. 17%) and a higher number of diseases (45% vs. 22% with ≥ 3 of diseases) compared to those without cancer. A significantly higher proportion of those with MI were female (60–61% vs. 51–52%), being a current smoker (20–25% vs. 11–13%) and obese (40–43% vs. 31–35%), and had a lower socioeconomic status (25–26% vs. 17–19% in decile 1–2) and a higher number of diseases (37–56% vs. 17–40% with ≥ 3 of diseases) compared to those without a MI in both the cancer and non-cancer groups.

**Table 1:** Characteristics of the study population by cancer and mental illness status

Characteristics, *n* (%)	Cancer			Non-cancer		
	Overall *n* = 2276	MI *n* = 690	No MI *n* = 1586	Overall *n* = 12,920	MI *n* = 3483	No MI *n* = 9437
Sex						
Female	1220 (54)	419 (61)	801 (51)	6962 (54)	2093 (60)	4869 (52)
Male	1056 (46)	271 (39)	785 (49)	5958 (46)	1390 (40)	4568 (48)
Age group in years						
25–34	56 (2)	18 (3)	38 (2)	2520 (20)	675 (19)	1845 (20)
35–49	241 (11)	108 (16)	133 (8)	4055 (31)	1105 (32)	2950 (31)
50–64	681 (30)	241 (35)	440 (28)	3456 (27)	996 (29)	2460 (26)
≥ 65	1298 (57)	323 (47)	975 (61)	2889 (22)	707 (20)	2182 (23)
Country of birth						
Australia	1801 (79)	561 (81)	1240 (78)	8652 (67)	2600 (75)	6052 (64)
Main English-speaking countries	275 (12)	77 (11)	198 (12)	1469 (11)	366 (10)	1103 (12)
Other	200 (9)	52 (8)	148 (9)	2799 (22)	517 (15)	2282 (24)
Geographical location						
Major cities	1279 (56)	402 (58)	877 (55)	7982 (62)	2106 (60)	5876 (62)
Inner regional	557 (24)	170 (25)	387 (24)	2546 (20)	741 (21)	1805 (19)
Other	440 (19)	118 (17)	322 (20)	2392 (18)	636 (18)	1756 (19)
Education level						
Postgraduate	189 (8)	44 (6)	145 (9)	1394 (11)	308 (9)	1086 (12)
Bachelor’s degree	279 (12)	79 (11)	200 (13)	2516 (19)	528 (15)	1988 (21)
Diploma	220 (10)	68 (10)	152 (10)	1447 (11)	408 (12)	1039 (11)
Certificate	534 (23)	164 (24)	370 (23)	2736 (21)	762 (22)	1974 (21)
No non-school qualification	964 (42)	306 (44)	658 (41)	4399 (34)	1364 (39)	3035 (32)
Level not determined	90 (4)	29 (4)	61 (4)	428 (3)	113 (3)	315 (3)
Socioeconomic status (index of relative socioeconomic disadvantage)						
Decile 1–2 (lowest)	475 (21)	178 (26)	297 (19)	2432 (19)	845 (25)	1587 (17)
Decile 3–4	533 (23)	170 (25)	363 (23)	2650 (21)	774 (22)	1876 (20)
Decile 5–6	440 (19)	130 (19)	310 (20)	2816 (22)	711 (20)	2105 (22)
Decile 7–8	427 (19)	118 (17)	309 (19)	2648 (20)	665 (19)	1983 (21)
Decile 9–10 (highest)	401 (18)	94 (14)	307 (19)	2374 (18)	488 (14)	1886 (20)
Smoker status						
Never smoked	1008 (44)	262 (38)	746 (47)	6501 (50)	1414 (41)	5087 (54)
Ex-smoker	952 (42)	287 (42)	665 (42)	4304 (33)	1205 (35)	3099 (33)
Current smoker	316 (14)	141 (20)	175 (11)	2115 (16)	864 (25)	1251 (13)
Physical activity						
High	225 (10)	50 (7)	175 (11)	2184 (17)	463 (13)	1721 (18)
Moderate	681 (30)	200 (29)	481 (30)	4390 (34)	1022 (29)	3368 (36)
Low-very low	834 (37)	258 (37)	576 (36)	4155 (32)	1225 (35)	2930 (31)
No physical activity/not stated	536 (23)	182 (26)	354 (22)	2191 (17)	773 (22)	1418 (15)
Alcohol consumption (lifetime alcohol risk level 7-day average 2009 guidelines)						
Exceeded guidelines	404 (18)	131 (19)	273 (17)	2226 (17)	622 (18)	1604 (17)
Did not exceed guidelines	878 (39)	233 (34)	645 (41)	4971 (38)	1192 (34)	3779 (40)
Last consumed 1 week to < 12 months ago	456 (20)	159 (23)	297 (19)	2941 (23)	868 (25)	2073 (22)
Last consumed ≥ 12 months ago	281 (12)	96 (14)	185 (12)	1157 (9)	404 (12)	753 (8)
Never consumed alcohol	202 (9)	55 (8)	147 (9)	1442 (11)	345 (10)	1097 (12)
Time since last consumed not known	55 (2)	16 (2)	39 (3)	183 (1)	52 (1)	131 (1)
Dietary intake (fruit and vegetables consumption guideline)						
Met both guidelines	170 (7)	43 (6)	127 (8)	738 (6)	182 (5)	556 (6)
Met vegetable guideline only	71 (3)	20 (3)	51 (3)	308 (2)	98 (3)	210 (2)
Met fruit guideline only	1082 (48)	294 (43)	788 (50)	5932 (46)	1424 (41)	4508 (48)
Did not meet either guideline	953 (42)	333 (48)	620 (39)	5942 (46)	1779 (51)	4163 (44)
Body mass index						
Underweight/normal	574 (25)	156 (23)	418 (26)	3950 (31)	971 (28)	2979 (32)
Overweight	849 (37)	239 (35)	610 (38)	4645 (36)	1126 (32)	3519 (37)
Obese	853 (37)	295 (43)	558 (35)	4325 (33)	1386 (40)	2939 (31)
Number of other diseases (excluding cancer and MI)						
0	247 (11)	49 (7)	198 (12)	3503 (27)	520 (15)	2983 (32)
1–2	1013 (44)	255 (37)	758 (48)	6553 (51)	1698 (49)	4855 (51)
3–4	789 (35)	266 (39)	523 (33)	2410 (19)	995 (29)	1415 (15)
≥ 5	227 (10)	120 (17)	107 (7)	454 (3)	270 (8)	184 (2)
Cancer disease status						
Current cancer	460 (20)	151 (22)	309 (19)	NA		
Non-current cancer	1816 (80)	539 (78)	1277 (81)			

*MI* mental illness

### Characteristics associated with MI

Several characteristics were associated with higher odds of MI in both the cancer and non-cancer groups: being female (aOR ranged 1.36–1.54), younger age (aOR ranged 2.10–3.67 for ages 35–49 compared to ≥ 65 years), obese (aOR ranged 1.14–1.30), current smoker (aOR ranged 1.66–2.17), having resided in major cities (aOR ranged 1.17–1.43), a lower socioeconomic status (aOR ranged 1.56–1.64 for decile 1–2) and physical activity level (aOR ranged 1.51–1.56 for no physical activity and 1.32–1.51 for low-very low physical activity compared to high level of physical activity), and a higher number of diseases (aOR ranged 2.50–4.63 for 3–4 number of diseases) (Table 2). The magnitude of the odds ratios associated with MI tended to be higher in those with cancer for nearly all characteristics, except for younger age, current smoker status and number of diseases where odds ratios were higher in the non-cancer group. Subjects born outside of Australia also had a lower odds of MI amongst those without cancer.

**Table 2:** Characteristics associated with mental illness (yes versus no) in cancer and non-cancer groups

Characteristics	Adjusted odds ratio (95% CI)	
	Cancer	Non-cancer
Sex		
Male	Reference	Reference
Female	1.54 (1.25–1.91)*	1.36 (1.24–1.48)*
Age group in years		
25–34	2.36 (1.26–4.39)*	2.47 (2.12–2.87)*
35–49	3.67 (2.62–5.13)*	2.10 (1.84–2.40)*
50–64	1.96 (1.56–2.47)*	1.61 (1.42–1.83)*
≥ 65	Reference	Reference
Country of birth		
Australia	Reference	Reference
Main English-speaking countries	1.00 (0.74–1.36)	0.81 (0.71–0.93)*
Other	0.80 (0.56–1.14)	0.59 (0.52–0.66)*
Geographical location		
Major cities	1.43 (1.16–1.76)*	1.17 (1.07–1.29)*
Inner regional/other	Reference	Reference
Education level		
Postgraduate	Reference	Reference
Bachelor’s degree	1.25 (0.79–1.98)	0.86 (0.73–1.02)
Diploma	1.19 (0.74–1.91)	1.07 (0.89–1.28)
Certificate	1.25 (0.82–1.91)	0.93 (0.79–1.10)
No non-school qualification	1.22 (0.81–1.84)	1.05 (0.90–1.23)
Level not determined	1.32 (0.73–2.40)	0.96 (0.73–1.25)
Socioeconomic status (index of relative socioeconomic disadvantage)		
Decile 1–2 (lowest)	1.64 (1.16–2.32)*	1.56 (1.34–1.80)*
Decile 3–4	1.39 (1.00–1.94)	1.36 (1.18–1.57)*
Decile 5–6	1.27 (0.91–1.79)	1.17 (1.02–1.35)*
Decile 7–8	1.13 (0.81–1.59)	1.18 (1.03–1.36)*
Decile 9–10 (highest)	Reference	Reference
Smoker status		
Never smoked	Reference	Reference
Ex-smoker	1.24 (0.99–1.54)	1.33 (1.21–1.47)*
Current smoker	1.66 (1.23–2.24)*	2.17 (1.93–2.45)*
Physical activity		
High	Reference	Reference
Moderate	1.59 (1.08–2.34)*	1.04 (0.91–1.19)
Low-very low	1.51 (1.02–2.22)*	1.32 (1.16–1.51)*
No physical activity/not stated	1.56 (1.03–2.37)*	1.51 (1.30–1.77)*
Alcohol consumption (lifetime alcohol risk level 7-day average 2009 guidelines)		
Exceeded guidelines	1.36 (1.02–1.80)*	1.12 (0.99–1.27)
Did not exceed guidelines	Reference	Reference
Last consumed 1 week to < 12 months ago	1.27 (0.98–1.66)	1.20 (1.07–1.34)*
Last consumed ≥ 12 months ago	1.16 (0.85–1.59)	1.47 (1.27–1.71)*
Never consumed alcohol	1.02 (0.70–1.49)	1.08 (0.92–1.26)
Time since last consumed not known	1.12 (0.59–2.11)	1.12 (0.79–1.59)
Dietary intake (fruit and vegetables consumption guideline)		
Met both guidelines	Reference	Reference
Met vegetable guideline only	0.96 (0.49–1.87)	1.12 (0.82–1.53)
Met fruit guideline only	1.12 (0.76–1.67)	0.96 (0.80–1.16)
Did not meet either guideline	1.47 (0.99–2.21)	1.09 (0.90–1.32)
Body mass index		
Underweight/normal	Reference	Reference
Overweight	1.15 (0.89–1.49)	%1.%2 (0.90–1.12)
Obese	1.30 (1.01–1.68)*	1.14 (1.02–1.27)*
Number of other diseases (excluding cancer and MI)		
0	Reference	Reference
1–2	1.53 (1.07–2.20)*	2.12 (1.90–2.38)*
3–4	2.50 (1.71–3.66)*	4.63 (4.03–5.31)*
≥ 5	5.98 (3.80–9.41)*	9.42 (7.50–11.84)*
Cancer disease status		NA
Non-current cancer	Reference	
Current cancer	1.10 (0.86–1.39)	

*CI* confidence interval, *MI* mental illness. *P < 0.05

### Health literacy

In the cancer group, those with a MI had a lower mean HL score across all the individual HL domains compared to those without a MI (Table 3). Similar trends were observed in the non-cancer group, except for one domain (domain 1: feeling understood and supported by healthcare providers). People with both cancer and MI had the lowest mean score in nearly all individual HL domains across the four groups, except for two domains (domain 1: feeling understood and supported by healthcare providers, and domain 3: actively managing my health) where they scored better than those with MI but no cancer. Domain 5 (appraisal of health information) had the lowest mean score from domains 1 to 5 (domains with four-point response options) across the four groups, whereas domain 9 (understand health information well enough to know what to do) recorded the highest score from domains 6 to 9 (domains with five-point response options).

**Table 3:** Health literacy domains mean scores by cancer and mental illness status

Health literacy domain	Cancer Mean score (SD)		Non-cancer Mean score (SD)		Δ, *p*-value		ꞵ, *p*-value^a^
	MI *n* = 289	No MI *n* = 666	MI *n* = 1284	No MI *n* = 3211	Main effect for cancer	Main effect for MI	Interaction effect for cancer X MI
Domain 1	3.19 (0.51)	3.26 (0.46)	3.18 (0.49)	3.17 (0.49)	Δ = −0.026,	Δ = 0.031,	ꞵ = −0.063,
Feeling understood and supported by healthcare providers					0.1933	0.1077	0.0957
Domain 2	3.09 (0.47)	3.17 (0.38)	3.10 (0.43)	3.20 (0.41)	Δ = 0.010,	Δ = 0.066,	ꞵ = 0.013,
Having sufficient information to manage my health					0.5371	< 0.0001*	0.6751
Domain 3	3.07 (0.42)	3.13 (0.41)	3.04 (0.46)	3.12 (0.45)	Δ = −0.011,	Δ = 0.028,	ꞵ = 0.030,
Actively managing my health					0.5104	0.1051	0.3661
Domain 4	3.04 (0.53)	3.17 (0.45)	3.05 (0.51)	3.18 (0.44)	Δ = −0.021,	Δ = 0.091,	ꞵ = 0.008,
Social support for health					0.2646	< 0.0001*	0.8213
Domain 5	2.85 (0.46)	2.93 (0.44)	2.89 (0.45)	2.94 (0.44)	Δ = −0.004,	Δ = 0.062,	ꞵ = −0.049,
Appraisal of health information					0.8327	0.0004*	0.1534
Domain 6	3.99 (0.77)	4.31 (0.57)	4.06 (0.68)	4.23 (0.58)	Δ = 0.039,	Δ = 0.203,	ꞵ = −0.112,
Ability to actively engage with healthcare providers					0.1187	< 0.0001*	0.0193*
Domain 7	3.85 (0.73)	4.15 (0.57)	3.89 (0.67)	4.06 (0.59)	Δ = 0.042,	Δ = 0.193,	ꞵ = −0.088,
Navigating the healthcare system					0.0894	< 0.0001*	0.0656
Domain 8	3.88 (0.71)	4.14 (0.55)	4.02 (0.59)	4.15 (0.56)	Δ = 0.060,	Δ = 0.160,	ꞵ = −0.125,
Ability to find good health information					0.0103*	< 0.0001*	0.0053*
Domain 9	4.14 (0.66)	4.33 (0.50)	4.21 (0.57)	4.32 (0.55)	Δ = 0.029,	Δ = 0.123,	ꞵ = −0.076,
Understand health information well enough to know what to do					0.1991	< 0.0001*	0.0744

*MI* mental illness, *SD* standard deviation. *p-value < 0.05. Δ = the difference between least square means for the effect of cancer (no cancer – cancer) and effects of MI (no MI – MI). Four response options for domains 1–5 and five response options for domains 6–9

^a^*P*-values come from the general liner models with main effects for cancer, MI, and an interaction for cancer X MI

MI status was associated with significant negative effects on seven individual HL domain scores (domain 2: having sufficient information to manage my health; domain 4: social support for health; domain 5: appraisal of health information; domain 6: ability to actively engage with healthcare providers; domain 7: navigating the healthcare system; domain 8: ability to find good health information; and domain 9: understand health information well enough to know what to do), such that those with MI had lower literacy levels than those without MI. Cancer status was associated with a significant negative effect on one domain only (domain 8: ability to find good health information), such that those with cancer had significantly lower literacy levels than those without cancer.

There were also significant interactions between cancer and MI status on two individual HL domain scores (domain 6: ability to actively engage with healthcare providers, and domain 8: ability to find good health information) that suggested there was not an additive effect for subjects with both diagnoses.

Among people in the cancer group, cancer disease status was associated with a significant negative effect on two domains (domain 2: having sufficient information to manage my health, and domain 9: understand health information well enough to know what to do), such that those with a current cancer disease status had significantly lower literacy levels than those with a non-current cancer disease status (Supplementary Table 1). MI status was associated with significant negative effects on six individual domain scores (domains 4 to 9), such that those with MI had lower literacy levels than those without MI within the cancer group.

## Discussion

This large cross-sectional study comprising over 15,000 people showed a high prevalence of MI in people both with and without cancer, with 30% and 27% of people with vs without cancer respectively having a current MI. The study identified that sociodemographic (sex, age, socioeconomic status, geographical location), lifestyle factors (smoker status, physical activity), and other conditions (obesity, number of diseases) increased the likelihood of having MI. Notably, in all but three of these characteristics, the magnitude of risk was higher in people with cancer. People with both cancer and MI had a lower HL in nearly all the individual HL domains compared to people without cancer or MI, people with MI but no cancer, and people with cancer but no MI. MI status alone was associated with significant negative effects on seven individual HL domain scores, whereas cancer status alone was associated with significant negative effect on one domain only. There were significant interaction effects between cancer and MI status on two domains that suggested the main effects were not additive for people with both diagnoses. Our findings provide insights into the risk factors for MI, and the association between cancer and MI status and HL to inform the development of targeted intervention strategies.

Our study found several sociodemographic characteristics including younger age, being female, a lower socioeconomic status and having resided in major cities to be linked with higher odds of MI in both cancer and non-cancer groups. It is plausible that younger participants may be more likely to identify themselves as having a mental health problem due to increased public health awareness campaigns over time including in schools [23, 24]. Although several systematic reviews have examined the risk factors for depression – the most common MI in cancer patients– and showed association between age and depression, the findings were inconclusive [10, 25, 26]. For example, of the 17 studies included in a systematic review that showed significant results, two studies reported middle-aged patients with cancer to be most affected by depression, seven studies found younger patients were more likely to be affected by depression than older patients, and the remaining eight studies found the opposite results [10]. In contrast, being female has been consistently identified as a risk factor for MI including depression [10, 27, 28] and anxiety [29, 30] in cancer and the general population, which may be due to sex differences in the disease prevalence, biological (e.g., hormones, genetics), psychological (personality and coping styles), and environmental factors (e.g., structural gender inequality) [27, 28]. The effects of socioeconomic status have also been examined with ten of the 25 studies included in a systematic review showing a lower socioeconomic status was associated with depression in cancer patients [10]. Geographical location may play a part whereby a meta-analysis showed that the pooled total prevalence rates for any psychiatric disorders were significantly higher in urban areas than rural areas [31], which may be due to interplay between social and environmental factors [32]. Taken together, our results suggest that these sociodemographic characteristics were risk factors for MI in general rather than cancer-specific findings, although the magnitude of risks tended to be higher in people with cancer.

Several lifestyle factors including being a current smoker and physically inactive, and the presence of other conditions including a higher number of diseases and obesity were identified to be associated with MI regardless of cancer status. Tobacco smoking is linked to changes in the brain structure and function and as a potential symptom or contributing factor to MI [33]. Previous studies also showed that people with MI were twice as likely to smoke as other people without MI [34, 35]. The evidence on the relationship between physical activity and mental wellbeing has been well established with studies consistently showing physical activity to be positively associated with increased mental wellbeing [36]. The relationship between mental and physical comorbidity also appears to be bidirectional: the risk of MI increases in people with long-term physical conditions, and people with several mental disorders also at risk of developing other physical comorbidity [37]. Further, a considerable proportion of people ranging from 25 to 45% with several MI are living with obesity [38, 39] which may be due to poor lifestyle practices and other biological links such as cell stress and inflammation [40]. People living with obesity also often experience weight stigma and discrimination which is known to have a negative impact on mental health [41]. Given that poor lifestyle practices particularly smoking and physical inactivity are the leading risk factors for premature death in people with MI [36, 42], our finding underscores the importance of optimal management of these lifestyle factors and physical-mental comorbidity to improve health outcomes in this vulnerable group of people.

While several associations between MI and HL domains reached statistical significance in our study, some differences in mean scores across groups appear relatively modest. For instruments such as the HL Questionnaire, mean differences of approximately 0.2–0.5 points per domain are generally interpreted as small-to-moderate and meaningful differences [43, 44], although no universal minimal clinically important difference has been established [45]. Our study found that HL was negatively associated with both MI and cancer, with stronger associations for MI. There is limited data on the association between the coexistence of both conditions and HL. A study conducted in Denmark comparing HL (covering only two domains of the HL Questionnaire; domain 6: the ability to actively engage with healthcare providers and domain 9: the ability to understand health information) between people with selected long-term health conditions including cancer and mental disorders and the general population [46]. The Danish study revealed that people with MI encountered more difficulties in actively engaging with healthcare providers than other long-term conditions, whereas people with cancer had fewer difficulties [46]. Presence of MI was also associated with greater difficulties in understanding health information. Our results concurred with these prior findings and further demonstrated that the interaction effects between cancer and MI status on two individual HL domain scores (including domain 6: ability to actively engage with healthcare providers) were less than additive for subjects with both diagnoses. Additionally, we examined all nine individual domains of the HL Questionnaire and found that MI status was associated with significant negative effects on seven individual HL domain scores, suggesting that this specific population may experience more HL challenges and require more support [47]. Potential reasons contributing to lower HL among people with MI include cognitive or functional impairments, socioeconomic disadvantage, system-level barriers (e.g., fragmented healthcare systems), and fear of discrimination which may lead to information avoidance [48, 49]. As people with MI are more likely to have high needs for health services but are more susceptible to inequalities (e.g., barriers to access to care) than those without MI [50], improvements in HL may have potential to alter health outcomes of this underserved population. Among people with cancer, having a current cancer disease status (versus non-current) was associated with significant negative effects on two individual HL domains scores (domain 2: having sufficient information to manage my health and domain 9: the ability to understand health information). Oncology patients are often required to process large amount of information about their care at time of cancer diagnosis, which is associated with significant stress [51]. Our findings highlight the importance of developing more specific and tailored interventions targeting these populations at greatest risk to improve their HL.

This study has several limitations. The cross-sectional design restricts the ability to infer the directionality of associations between MI and cancer status and HL. The study was also limited by self-reported nature of the data collected in the surveys which may subject to response or recall bias. MI may be underreported due to stigma associated with MI which may lead to nondisclosure among respondents. While we were able to differentiate cancer disease status according to respondents’ self-reported condition status indicating whether their cancer was current or non-current, we were not able to account for duration of disease, cancer types or stages as this information was not captured in the dataset. Further, we grouped individuals with any type of MI into a single MI category; however, health literacy and modifiable risk factors may differ across specific mental health conditions, which was not accounted for in this study. Due to small sample size of specific MI, it was not possible to conduct subgroup analyses, and that future epidemiologic studies could usefully explore this. HL is also likely to change over time according to an individual’s health conditions, health status and their current needs and encounters with health services. Therefore, further longitudinal data is required to fully capture an individual’s HL journey and the potential influence of health conditions on HL and health outcomes.

Despite these limitations, the study’s findings are highly useful for designing targeted interventions to improve HL because they identify specific modifiable risk factors and vulnerable populations. The study established that several modifiable risk factors (obesity, smoking, physical inactivity) are associated with higher odds of MI, and MI is strongly and negatively associated with seven out of nine HL domains. This suggests a dual-pronged approach for interventions including targeting modifiable risk factors with HL in mind. For example, the support program (e.g., smoking cessation, exercise program, nutrition and weight management) could be delivered in a way that is easy to understand, addressing domains where literacy is lowest, such as appraising health information (Domain 5). The second approach to creating interventions would be to directly address specific HL deficits. For example, the study found that people MI (with or without cancer) had significant difficulties in domains like "navigating the healthcare system" (Domain 7), "finding good health information" (Domain 8) and “understand health information well” (Domain 9). Therefore, an intervention targeting the healthcare system could be a patient navigator program, where a dedicated support person helps individuals schedule appointments, and find reliable online resources. Healthcare systems and providers could tailor communication strategies and incorporate visual aids to address varying levels of HL. This would directly support the specific literacy domains where this population struggles most.

## Conclusions

In conclusion, risk factors for MI appeared to be similar in persons with and without cancer, but the associated relative risks are higher in those with cancer. HL is negatively associated with both MI and cancer, with stronger associations for MI. Our research suggests that interventions should not just focus on improving HL in isolation. Instead, a more effective strategy would be to integrate HL support into programs that simultaneously address the modifiable risk factors for MI, thereby improving the overall outcomes for this vulnerable population.

## Supplementary Information

Below is the link to the electronic supplementary material.

ESM 1 (DOCX 34.4 KB)

## Data Availability

With appropriate approvals, the data may be accessed through the Australian Bureau of Statistics.
