# Supplementary material for: Mental illness and health literacy in people with cancer: the Australian National Health Survey analysis
Source: Support Care Cancer. 2026 Jul 2;34(7):722. doi: 10.1007/s00520-026-10962-x (PMC13328223; doi:10.1007/s00520-026-10962-x)
Supplement: Supplementary file 1 — (DOCX 34.4 KB) [file 520_2026_10962_MOESM1_ESM.docx]

**Supplementary Table 1** Health literacy domains mean scores by cancer disease status (current versus non-current) and mental illness status among those with a history of cancer

| **Health literacy domain** | **Current cancer**  **Mean score (SD)** | | **Non-current cancer**  **Mean score (SD)** | | **Δ,** p**-value** | | **ꞵ, p-value^a^** |
| --- | --- | --- | --- | --- | --- | --- | --- |
|  | **MI**  **n=64** | **No MI**  **n=125** | **MI**  **n=225** | **No MI**  **n=541** | **Main effect for cancer status** | **Main effect for MI** | **Interaction effect for cancer X MI** |
| **Domain 1**  Feeling understood and supported by healthcare providers | 3.19 (0.48) | 3.29 (0.47) | 3.19 (0.52) | 3.26 (0.46) | Δ=0.044,  0.2853 | Δ=0.041,  0.3360 | ꞵ=-0.021,  0.7973 |
| **Domain 2**  Having sufficient information to manage my health | 2.99 (0.46) | 3.11 (0.36) | 3.12 (0.47) | 3.19 (0.39) | Δ=-0.078,  0.0305* | Δ=0.062,  0.0947 | ꞵ=0.022,  0.7627 |
| **Domain 3**  Actively managing my health | 3.06 (0.43) | 3.07 (0.43) | 3.08 (0.41) | 3.14 (0.40) | Δ=-0.030,  0.3937 | Δ=-0.001,  0.9718 | ꞵ=-0.032,  0.6528 |
| **Domain 4**  Social support for health | 3.01 (0.51) | 3.17 (0.41) | 3.05 (0.53) | 3.17 (0.45) | Δ=0.002,  0.9591 | Δ=0.088,  0.0422* | ꞵ=0.034,  0.6868 |
| **Domain 5**  Appraisal of health information | 2.79 (0.53) | 2.86 (0.40) | 2.86 (0.44) | 2.94 (0.44) | Δ=-0.066,  0.0897 | Δ=0.080,  0.0463* | ꞵ=-0.005,  0.9503 |
| **Domain 6**  Ability to actively engage with healthcare providers | 3.89 (0.88) | 4.25 (0.56) | 4.02 (0.73) | 4.32 (0.57) | Δ=-0.099,  0.0801 | Δ=0.283,  <0.0001* | ꞵ=0.064,  0.5673 |
| **Domain 7**  Navigating the healthcare system | 3.85 (0.77) | 4.11 (0.55) | 3.85 (0.72) | 4.15 (0.58) | Δ=-0.015,  0.7810 | Δ=0.210,  0.0002* | ꞵ=-0.068,  0.5351 |
| **Domain 8**  Ability to find good health information | 3.78 (0.86) | 4.06 (0.51) | 3.91 (0.67) | 4.16 (0.56) | Δ=-0.094,  0.0756 | Δ=0.236,  <0.0001* | ꞵ=0.040,  0.7058 |
| **Domain 9**  Understand health information well enough to know what to do | 3.99 (0.84) | 4.27 (0.50) | 4.18 (0.60) | 4.35 (0.51) | Δ=-0.128,  0.0092* | Δ=0.227,  <0.0001* | ꞵ=0.124,  0.2029 |

Key: MI, mental illness; SD, standard deviation. *p-value<0.05. Δ = the difference between least square means for the effect of cancer (current cancer – non-current cancer) and effects of MI (no MI – MI). Four response options for domains 1-5 and five response options for domains 6-9.

^a^P-values come from the general liner models with main effects for cancer, MI, and an interaction for cancer X MI.
